# HDL functionality and cardiovascular outcome among nondialysis chronic kidney disease patients[S]

**DOI:** 10.1194/jlr.P085076

**Published:** 2018-05-22

**Authors:** Kathrin Untersteller, Sabine Meissl, Markus Trieb, Insa E. Emrich, Adam M. Zawada, Michael Holzer, Eva Knuplez, Danilo Fliser, Gunnar H. Heine, Gunther Marsche

**Affiliations:** Internal Medicine IV–Nephrology and Hypertension,* Saarland University Medical Center, Homburg, Germany; Otto Loewi Research Center, Division of Pharmacology,† Medical University of Graz, Graz, Austria; BioTechMed-Graz, § Graz, Austria

**Keywords:** HDL function, paraoxonase activity, cholesterol efflux capacity, antioxidative activity, cardiovascular events

## Abstract

CVD remains the leading cause of morbidity and mortality in patients with chronic kidney disease (CKD). CKD profoundly affects HDL composition and functionality, but whether abnormal HDL independently contributes to cardiovascular events in CKD patients remains elusive. In the present study, we assessed whether compositional and functional properties of HDL predict cardiovascular outcome among 526 nondialysis CKD patients who participate in the CARE FOR HOMe study. We measured HDL cholesterol, the content of HDL-associated proinflammatory serum amyloid A (SAA), and activities of the HDL enzymes paraoxonase and lipoprotein-associated phospholipase A2 (Lp-PLA_2_). In addition, we assessed the antioxidative activity of apoB-depleted serum. During a mean follow-up of 5.1 ± 2.1 years, 153 patients reached the predefined primary endpoint, a composite of atherosclerotic cardiovascular events including cardiovascular mortality and death of any cause. In univariate Cox regression analyses, lower HDL-cholesterol levels, higher HDL-associated SAA content, and lower paraoxonase activity predicted cardiovascular outcome, while Lp-PLA_2_ activity and antioxidative capacity did not. HDL-cholesterol and HDL-paraoxonase activity lost their association with cardiovascular outcome after adjustment for traditional cardiovascular and renal risk factors, while SAA lost its association after further adjustment for C-reactive protein. In conclusion, our data suggest that neither HDL quantity nor HDL composition or function independently predict cardiovascular outcome among nondialysis CKD patients.

There is unequivocal evidence of an inverse association between plasma HDL cholesterol (HDL-C) concentrations and the risk of CVD, a finding that has led to the hypothesis that HDL protects from CVD. In line with the HDL hypothesis, HDL exhibits many potential antiatherogenic properties (1). HDL can act as an acceptor of cellular cholesterol, which constitutes the first step in a pathway known as reverse cholesterol transport (2). In addition, HDL isolated from healthy subjects shows potent antiinflammatory and antioxidant properties (3). apoA-I is the most important structural protein of HDL with antioxidant properties (4, 5). Consequently, HDL has the capacity to inhibit the oxidative modification of LDL and thereby reduce the atherogenicity of these lipoproteins (4). HDL-associated paraoxonase shows antiinflammatory (6, 7) and endothelial protective activities (8).

Chronic kidney disease (CKD) profoundly alters enzyme activities involved in HDL metabolism, affecting particle maturation and remodeling, thereby changing HDL composition and function (9–11). These compositional alterations include enrichment of uremic HDL with proinflammatory acute-phase protein serum amyloid A (SAA) and lipoprotein-associated phospholipase A2 (Lp-PLA_2_) content (12, 13), whereas antiinflammatory HDL-paraoxonase content and activity are markedly reduced (11).

Of note, despite substantial experimental work on HDL in CKD, the clinical implications of most of these structural and functional alterations of HDL have remained enigmatic up to now. Earlier work on HDL functionality largely focused on its promotion of cholesterol efflux from macrophages (HDL-C efflux capacity). In the general population (14, 15) and among individuals at elevated cardiovascular risk (16), HDL cholesterol efflux capacity was inversely associated with incident cardiovascular events, even after adjusting for HDL-C levels. Importantly, these associations were not observed among CKD patients within the CARE FOR HOMe (Cardiovascular and Renal Outcome in CKD 2-4 Patients–The Fourth Homburg evaluation) (17) and the German Diabetes Dialysis Study (4D Study) (18). Given that oxidative stress and a chronic inflammatory state are major nontraditional risk factors among CKD patients (19–22), tests of antiinflammatory properties of HDL and antioxidative activity of serum may prove useful.

In the present study, we investigated whether metrics of HDL composition and function predict future cardiovascular events in a cohort of nondialysis CKD patients.

## MATERIALS AND METHODS

### Study design and patients

CARE FOR HOMe is an ongoing cohort study including patients with CKD G2–G4 according to the Modification of Diet in Renal Disease equation [estimated glomerular filtration rate (eGFR) 15–89 ml/min/1.73 m2] at baseline (23). CARE FOR HOMe excluded transplant recipients, pregnant women, patients under the age of 18, receivers of systemic immunosuppressive medication, and patients infected with human immunodeficiency virus. Clinically apparent infections [defined as C-reactive protein (CRP) levels > 50 mg/l, and/or requiring systemic antibiotic therapy] or active malignancy at baseline were excluded. Finally, patients with acute kidney injury were excluded.

All procedures involving human subjects were approved by the local Ethics Committee and carried out in accordance with the Code of Ethics of the World Medical Association (Declaration of Helsinki). Informed consent was obtained from all patients.

At baseline, all patients provided a fasting blood and a spot urine sample. A standardized questionnaire was used to collect data, including medical history, current medication, smoking status, and prevalent diabetes mellitus. Prevalent CVD was defined as any of the following: history of myocardial infarction, coronary artery angioplasty/stenting/bypass surgery, major stroke, carotid endarterectomy/stenting, nontraumatic lower extremity amputation, or lower limb artery angioplasty/stenting/bypass surgery. All prevalent cardiovascular events were confirmed by chart review.

Patients with self-reported or physician-reported diabetes mellitus, with a fasting blood glucose level of >126 mg/dl or treatment with hypoglycemic medication, were categorized as diabetic. Patients were defined as active smokers if they smoked at study start or had stopped smoking <1 month before entry into the study. Only 10 women reported estrogen treatment.

### Outcome analyses

Annually, we invited all patients to our study center for a follow-up examination, in which we collected information on cardiovascular events by a standardized questionnaire. Patients who became dialysis-dependent during follow-up and patients who refused to attend the regular follow-up visit were contacted by telephone, and laboratory information was provided from their treating primary care physician or their nephrologist. Two independent physicians adjudicated all events; in case of disagreement, a third physician was consulted.

The primary atherosclerotic cardiovascular endpoint was defined as the first occurrence of any of the following: myocardial infarction, coronary artery angioplasty/stenting/bypass surgery, major stroke, carotid endarterectomy/stenting, nontraumatic lower extremity amputation, and/or lower limb artery angioplasty/stenting/bypass surgery, or death of any cause.

As additional endpoints, we defined all-cause death as well as hospital admission for heart failure.

All cardiovascular events were confirmed by chart review.

### Laboratory measurements

A total of 544 patients were included into CARE FOR HOMe between 2008 and 2015. Serum was available from 526 patients for SAA, antioxidative activity, paraoxonase activity, Lp-PLA_2_ activity, HDL-C efflux capacity, and HDL-C measurements. The samples analyzed for these variables were drawn at baseline.

### SAA quantification

SAA content was quantified by ELISA (human SAA, BioSource Europe S.A., Belgium) as described (24, 25).

### Preparation of apoB-depleted serum

ApoB-depleted serum was prepared by the addition of 40 μl of polyethylene glycol (20% in 200 mmol/l glycine buffer) to 100 μl of serum. Samples were incubated for 20 min, and the supernatants were recovered after centrifugation (9,703 *g*, 20 min, 4°C) as described (26, 27). Subsequently, the HDL-containing supernatants (apoB-depleted serum) were collected and stored at –80°C.

### Antioxidative capacity

The antioxidative activity of apoB-depleted serum was determined as previously described (24, 27). Briefly, dihydrorhodamine (DHR) was suspended in DMSO to a 50 mmol/l stock, which was diluted in HEPES (20 mmol/l HEPES, 150 mmol/l NaCl, pH 7.4) containing 1 mmol/l 2,2’-azobis-2-methyl-propanimidamide-dihydrochloride to a 50 µmol/l working reagent. One microliter of apoB-depleted serum was placed into 384-well plates, 15 µl of DHR working reagent was added, and the volume was completed to 100 µl with HEPES buffer. The increase in fluorescence due to the oxidation of DHR was measured every 2 min for 1 h at 538 nm. The increase in fluorescence per minute was determined for samples containing only DHR and for samples containing DHR and individual serum samples.

### Paraoxonase activity

Ca^2+^-dependent arylesterase activity of paraoxonase was determined by a photometric assay using phenylacetate as substrates as described (25, 27). ApoB-depleted serum was added to 200 μl of buffer containing 100 mmol/l Tris, 2 mmol/l CaCl_2_ (pH 8.0), and 1 mmol/l phenylacetate. The rate of hydrolysis of phenylacetate was monitored by the increase of absorbance at 270 nm, and readings were taken every 15 s at room temperature to generate a kinetic plot. The slope from the kinetic chart was used to determine ΔAb270 nm/min. Enzymatic activity was calculated with the Beer–Lambert law from the molar extinction coefficient of 1,310 l·mol^−1^·cm^−1^.

### Lp-PLA_2_ activity

HDL-associated Lp-PLA_2_ activity was measured in apoB-depleted serum by using a commercially available photometric assay (Cayman Europe, Talinn, Estonia) using 2-thio platelet activating factor as substrate as described previously (24).

### Cholesterol efflux capacity

We quantified cholesterol efflux capacity with use of a previously published protocol (26–28). J774 cells, a mouse macrophage cell line, were plated and labeled for 24 h with 1 μCi/ml [^3^H]cholesterol (Perkin Elmer, Boston, MA). J774 cells express very low levels of ATP-binding cassette transporter A1 (ABCA1), an important pathway of cholesterol efflux from macrophages. To upregulate ABCA1, cells were stimulated for 6 h with serum-free DMEM containing 0.3 mmol/l 8-(4-chlorophenylthio)-cyclic AMP (Sigma, Darmstadt, Germany). After labeling, cells were washed, and [^3^H]cholesterol efflux was determined by incubating cells for 4 h with 2.8% apoB-depleted serum as described (26). The quantity of [^3^H]cholesterol incorporated into cellular lipids was assessed by means of isopropanol extraction of [^3^H]cholesterol content of J774 cells not exposed to the patients’ serum. All steps were performed in the presence of a concentration of 2 μg/ml of the acyl CoA cholesterol acyltransferase inhibitor Sandoz 58-035 (Sigma, Darmstadt, Germany). All assays were performed twice, and samples were assessed in duplicates. To correct for interassay variation across plates, we included a serum control on each plate, and we normalized values for serum samples from patients to this value in subsequent analyses. Across the entire cohort, duplicate measures of efflux capacity were highly correlated (*r* = 0.91).

### Statistical analyses

Categorical variables are presented as percentage of patients and compared by using the chi^2^ test. Continuous data are expressed as means ± SD, or median (interquartile ranges), as appropriate, and were compared by using one-way ANOVA test partitioning the between-groups sums of squares into trend components; continuous variables are presented as mean ± SD, or median (interquartile range) in case of skewed distribution. We calculated univariate correlation analyses using Spearman coefficients. For outcome analyses, we first performed Kaplan-Meier analysis with consecutive log-rank testing, after stratifying patients into tertiles by their HDL-C, SAA, antioxidative activity, paraoxonase, and Lp-PLA_2_ activities. Subsequently, we calculated univariate and multivariate Cox models to assess the association of *a*) HDL-C, *b*) logarithmized SAA (logSAA), *c*) antioxidative activity, *d*) paraoxonase activity, and *e*) Lp-PLA_2_ activity and cardiovascular outcome. All variables were considered both as continuous variables and as categorized variables, after stratification of patients into tertiles. We predefined multivariate Cox models with adjustment for age, gender, body mass index, mean blood pressure, smoking status, diabetes mellitus, eGFR, and log-transferred albuminuria (model 2), with additional adjustment for total cholesterol and HDL-C (for analyses with SAA, antioxidative activity, paraoxonase activity, and Lp-PLA_2_ activity as exposition variable) or log-transformed SAA, respectively (for analyses with HDL-C as exposition variable; model 3), and with additional adjustment for CRP (model 4).

## RESULTS

### Baseline characteristics of study participants

**Fig. 1.**
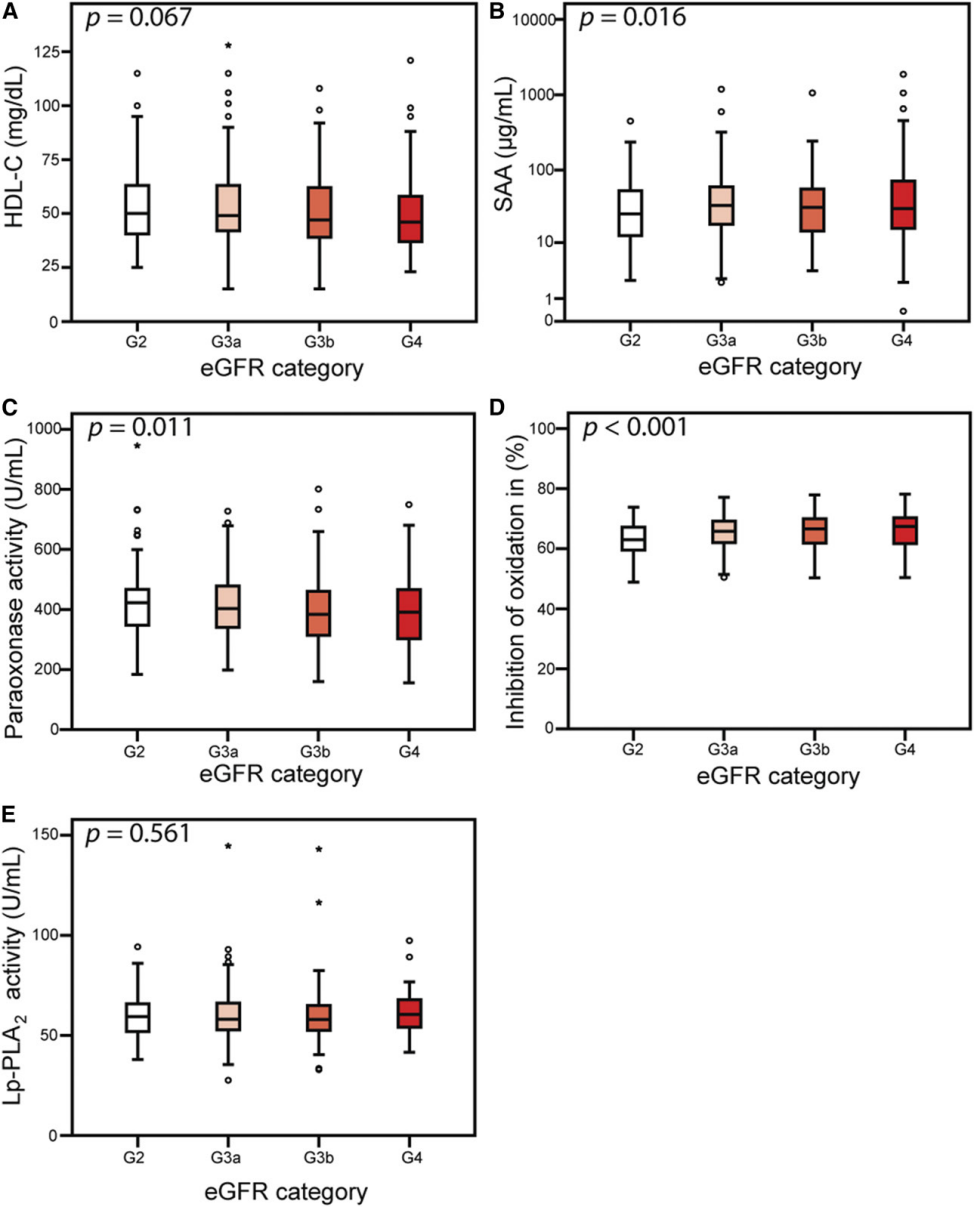
Parameters of cholesterol metabolism and renal function. Levels of HDL-C (A), SAA (B), paraoxonase activity (C), antioxidative activity (D), and Lp-PLA_2_ (E) stratified by eGFR category. Depicted are medians, interquartile ranges, and outliers.

The CARE FOR HOMe consists of 526 study patients stratified by Kidney Disease: Improving Global Outcomes stages. The mean age at study entry was 65.1 ± 12.3 years with a mean eGFR of 46 ± 16 ml/min/1.73 m^2^ (**Table 1**). Of those, 39% of all patients had prevalent CVD, and about one-third had diabetes mellitus. With declining renal function, people were older and had more cardiovascular comorbidities (Table 1). Serum samples of study subjects with more advanced CKD stages showed similar HDL-C levels (**Fig. 1A**), increased levels of the HDL associated acute phase protein SAA (*P* = 0.016) (Fig. 1B), higher antioxidative activity (*P* < 0.001) (Fig. 1C), and lower paraoxonase activity (*P* = 0.011) (Fig. 1D), whereas HDL-associated Lp-PLA_2_ activity was not altered (Fig. 1E). In addition, we stratified the study participants by the presence of diabetes mellitus (supplemental. Fig. S1). CKD patients with diabetes mellitus showed lower HDL-C levels (*P* < 0.001), increased SAA (*P* = 0.045), lower arylesterase activity of paraoxonase (*P* = 0.013), and HDL associated Lp-PLA_2_ activity (*P* = 0.010) whereas antioxidative activity was not different (*P* = 0.935).

**TABLE 1.** Baseline characteristics, stratified by eGFR categories

	CKD G 2 (n = 112)	CKD G 3a (n = 178)	CKD G 3b (n = 141)	CKD G 4 (n = 95)	Total cohort (n = 526)	*p*-values
Age (years)	58.8 ± 12.1	65.1 ± 12.5	68.3 ± 10.9	68.1 ± 11.4	65.1 ± 12.3	≤0.001
Sex (woman)	38 (34%)	81 (46%)	61 (43%)	38 (40%)	218 (41%)	0.252
eGFR (ml/min/1.73 m^2^)	69 ± 7	52 ± 4	38 ± 4	23 ± 4	46 ± 16	≤0.001
BMI (kg/m^2^)	30.4 ± 5.4	30.7 ± 5.5	30.6 ± 6.0	29.5 ± 4.7	30.4 ± 5.5	0.280
Diabetes mellitus (yes)	40 (36%)	69 (39%)	54 (38%)	40 (42%)	203 (39%)	0.827
Mean BP (mmHg)	108 ± 14	110 ± 14	105 ± 15	107 ± 16	108 ± 15	0.104
Systolic BP (mmHg)	149 ± 20	153 ± 24	150 ± 24	155 ± 27	152 ± 24	0.396
Diastolic BP (mmHg)	108 ± 14	110 ± 14	105 ± 15	107 ± 16	108 ± 15	≤0.001
Prevalent CVD (yes)	18 (16%)	55 (31%)	61 (43%)	31 (33%)	165 (31%)	≤0.001
Current nicotine (yes)	19 (17%)	15 (8%)	12 (9%)	8 (8%)	54 (10%)	0.074
CRP (mg/l)	2.35 (1.23–4.30)	2.5 (1.05–4.90)	2.9 (1.2–6.4)	3.3 (1.1–6-1)	2.7 (1.1–5.1)	0.001
Triglycerides (mg/dl)	157 ± 124	159 ± 116	165 ± 104	176 ± 125	163 ± 117	0.210
Total cholesterol (mg/dl)	195 ± 39	192 ± 43	189 ± 44	190 ± 47	192 ± 43	0.290
LDL-C (mg/dl)	118 ± 35	114 ± 37	112 ± 36	114 ± 39	114 ± 36	0.303
HDL-C (mg/dl)	53 ± 16	53 ± 18	51 ± 17	49 ± 17	52 ± 17	0.067
Lipid-lowering drugs (yes)	47 (42%)	100 (56%)	87 (62%)	52 (55%)	286 (54%)	0.017
Statins (yes)	43 (38%)	94 (53%)	84 (60%)	50 (53%)	271 (52%)	0.009
Lipid-lowering therapy other than statins (yes)	14 (13%)	16 (9%)	19 (14%)	7 (7%)	56 (11%)	0.365
ApoA-I (mg/dl)	166.3 ± 30.6	168.1 ± 31.0	164.5 ± 31.3	162.1 ± 35.5	165.7 ± 31.9	0.222
ApoB (mg/dl)	98.7 ± 24.6	100.3 ± 26.4	98.0 ± 25.4	99.0 ± 26.4	99.1 ± 25.7	0.814
Paraoxonase activity (U/ml)	423.43 ± 118.45	413.21 ± 104.76	388.36 ± 112.51	392.87 ± 122.51	405.05 ± 113.69	0.011
SAA (μg/ml)	25.46 (12.61–52.68)	33.13 (18.16–59.36)	31.13 (14.44–58.53)	30.15 (15.79–71.38)	29.95 (14.96–61.75)	0.016
Antioxidative activity (Inhibition of oxidation in %)	63.06 ± 5.29	65.34 ± 5.48	66.01 ± 5.95	66.03 ± 5.89	65.16 ± 5.74	≤0.001
Lp-PLA_2_ activity (U/ml)	59.55 ± 10.31	60.02 ± 11.97	59.25 ± 13.04	60.99 ± 9.92	59.89 ± 11.58	0.561

Values are means ± SD or interquartile ranges (in parentheses). BP, blood pressure; LDL-C, LDL cholesterol.

#### Association of CKD stages with markers of HDL functionality.

Kidney function weakly correlated with lower CRP (*P* = 0.031), higher HDL-C (*P* = 0.030), and higher paraoxonase activity (*P* = 0.014) (**Table 2**). Interestingly, kidney function was inversely associated with cholesterol efflux capacity (*P* = 0.006), suggesting that some metrics of HDL function are not affected and even improved in more advanced CKD stages. Moreover, kidney function was inversely associated with antioxidative activity of apoB-depleted serum (*P* < 0.001). The systemic inflammation marker CRP significantly correlated with lower HDL-C (*P* = 0.001), Lp-PLA_2_ activity (*P* = 0.001), and cholesterol efflux capacity (*P* < 0.001). As expected, a robust association between the systemic inflammation markers CRP and the HDL-associated SAA (*P* < 0.001) was observed. Further details and correlations between markers of HDL functionality are shown in Table 2.

**TABLE 2.** Univariate Spearman correlation coefficients

	eGFR		CRP		HDL-C		Paraoxonase activity		SAA		Antioxidative activity		Lp-PLA_2_ activity	
	Rho	*P*	Rho	*P*	Rho	*P*	Rho	*P*	Rho	*P*	Rho	*P*	Rho	*P*
CRP	−0.094	**0.031**	—	—	—	—	—	—	—	—	—	—	—	—
HDL-C	0.095	**0.030**	−0.193	**<0.001**	—	—	—	—	—	—	—	—	—	—
Paraoxonase activity	0.107	**0.014**	−0.067	0.123	0.245	**<0.001**	—	—	—	—	—	—	—	—
SAA	−0.073	0.092	0.575	**<0.001**	0.080	0.065	0.026	0.557	—	—	—	—	—	—
Antioxidative activity	−0.233	**<0.001**	0.042	0.342	−0.124	**0.004**	0.128	**0.003**	−0.043	0.321	—	—	—	—
Lp-PLA_2_ activity	−0.035	0.428	−0.139	**0.001**	0.075	0.085	0.136	**0.002**	−0.111	**0.011**	0.088	**0.045**	—	—
Cholesterol efflux capacity	−0.120	**0.006**	−0.148	**0.001**	0.497	**<0.001**	0.289	**<0.001**	−0.014	0.751	0.070	0.111	0.153	**<0.001**

Boldface type indicates significance (*P* < 0.05).

### Outcome analyses

**Fig. 2.**
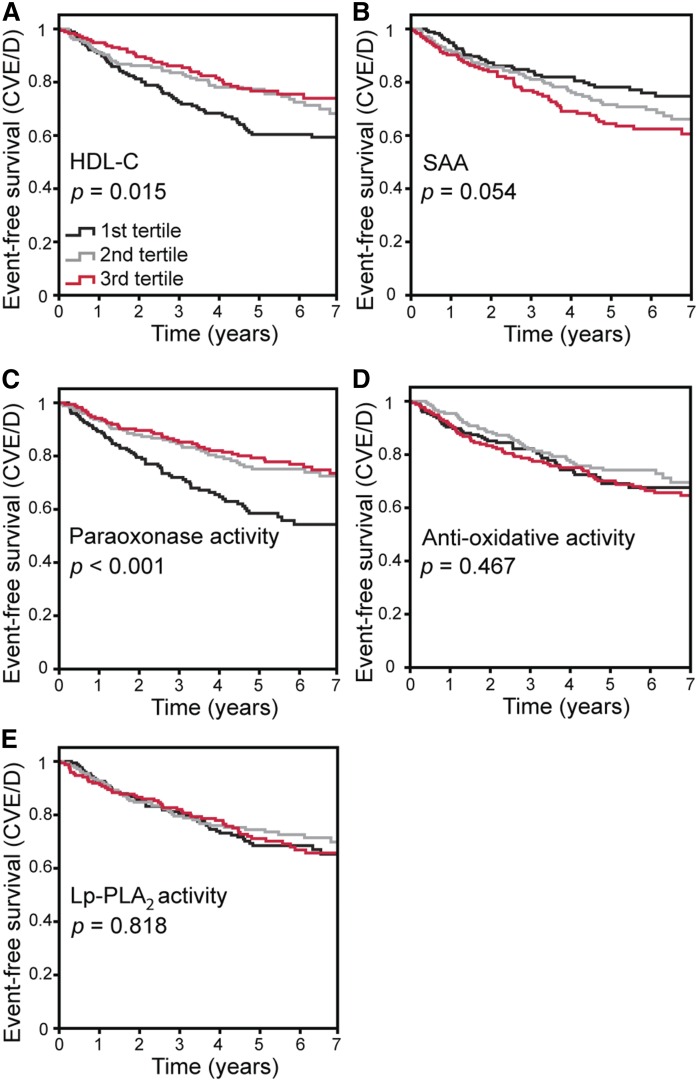
Kaplan-Meier analyses with subsequent log-rank test [endpoint cardiovascular events/death (CVE/D)]-event-free survival in CKD patients stratified by HDL-C (A), SAA (B), paraoxonase activity (C), antioxidative activity (D), and Lp-PLA_2_ (E).

During a mean follow-up of 5.1 ± 2.1 years, 153 patients reached the primary cardiovascular endpoint. After stratifying patients in tertiles for levels of markers of HDL-C quantity and functionality, lower levels of HDL-C (*P* = 0.015) and paraoxonase activity (*P* < 0.001) were associated with the occurrence of the primary endpoint in Kaplan-Meier analyses (**Fig. 2**). The primary endpoint was neither predicted by antioxidative activity (*P* = 0.467) nor by Lp-PLA_2_ (*P* = 0.818) nor by SAA (*P* = 0.054; Fig. 2). As additional endpoints, we defined all-cause death (which is majorly driven by cardiovascular death), as well as hospital admission for heart failure. We recalculated our analyses with these alternative endpoints, but did not find a substantial difference in the main results (supplemental Figs. S2 and S3).

Consistently, in univariate Cox regression analyses with HDL-C quantity and function markers considered as continuous variables, lower HDL-C and lower paraoxonase activity, but also higher logSAA, were predictors of adverse outcome (**Table 3**). After adjustment for traditional cardiovascular and renal risk factors, logSAA remained a significant predictor of the primary endpoint (*P* = 0.030; model 2), whereas HDL-C and paraoxonase activity did not. The association of logSAA with the primary endpoint continued to be significant after further adjustment for total cholesterol and HDL-C (*P* = 0.034; model 3), but did not persist after further adjustment for CRP (*P* = 0.935; model 4).

**TABLE 3.** Cox models (end-point cardiovascular events/all cause death)

	Model 1		Model 2		Model 3		Model 4	
	HR (CI 95%)	*P*	HR (CI 95%)	*P*	HR (CI 95%)	*P*	HR (CI 95%)	*P*
Continuous predictors								
HDL-C (mg/dl)	0.987 (0.976–0.997)	**0.014**	1.001 (0.990–1.013)	0.836	1.006 (0.994–1.018)	0.334	1.002 (0.990–1.013)	0.749
logSAA (μg/ml)	1.699 (1.201–2.402)	**0.003**	1.443 (1.037–2.009)	**0.030**	1.416 (1.026–1.954)	**0.034**	1.018 (0.666–1.556)	0.935
Paraoxonase activity (U/ml)	0.997 (0.995–0.998)	**<0.001**	0.999 (0.998–1.001)	0.309	1.000 (0.998–1.001)	0.760	1.000 (0.998–1.002)	0.899
Antioxidative activity (inhibition of oxidation, %)	1.014 (0.985–1.045)	0.340	0.997 (0.967–1.028)	0.837	1.002 (0.972–1.033)	0.885	1.007 (0.976–1.039)	0.664
Lp-PLA_2_ activity (U/ml)	0.997 (0.984–1.011)	0.688	1.003 (0.990–1.017)	0.629	1.009 (0.996–1.022)	0.196	1.010 (0.997–1.023)	0.133
Categorized predictors								
HDL-C								
1^st^ tertile		**0.017**		0.276		0.308		0.316
2^nd^ tertile	0.682 (0.469–0.991)	**0.045**	0.739 (0.499–1.094)	0.131	0.750 (0.505–1.112)	0.152	0.786 (0.530–1.165)	0.230
3^rd^ tertile	0.587 (0.395–0.871)	**0.008**	0.970 (0.499–1.094)	0.895	0.967 (0.614–1.525)	0.886	1.071 (0.675–1.699)	0.771
SAA								
1^st^ tertile		0.051		0.079		0.052		0.423
2^nd^ tertile	1.283 (0.851–1.936)	0.234	1.526 (1.006–2.314)	**0.047**	1.562 (1.029–2.372)	**0.036**	1.323 (0.850–2.060)	0.215
3^rd^ tertile	1.633 (1.099–2.428)	**0.015**	1.512 (1.007–2.269)	**0.046**	1.587 (1.053–2.392)	**0.027**	1.123 (0.673–1.873)	0.658
Paraoxonase activity								
1^st^ tertile		**<0.001**		0.790		0.613		0.570
2^nd^ tertile	0.549 (0.375–0.806)	**0.002**	0.874 (0.584–1.308)	0.512	0.940 (0.625–1.414)	0.768	1.040 (0.686–1.578)	0.853
3^rd^ tertile	0.477 (0.323–0.704)	**<0.001**	0.905 (0.597–1.370)	0.636	1.173 (0.751–1.830)	0.483	1.259 (0.803–1.946)	0.316
Antioxidative activity								
1^st^ tertile		0.406		0.583		0.623		0.367
2^nd^ tertile	0.818 (0.542–1.235)	0.340	0.806 (0.531–1.222)	0.309	0.814 (0.536–1.235)	0.333	0.756 (0.497–1.150)	0.191
3^rd^ tertile	1.058 (0.719–1.557)	0.774	0.864 (0.580–1.288)	0.473	0.915 (0.613–1.367)	0.666	0.956 (0.640–1.428)	0.827
Lp-PLA_2_ activity								
1^st^ tertile		0.852		0.634		0.353		0.210
2^nd^ tertile	0.896 (0.606–1.324)	0.581	1.218 (0.812–1.828)	0.340	1.280 (0.852–1.922)	0.235	1.384 (0.919–2.086)	0.120
3^rd^ tertile	0.929 (0.632–1.365)	0.707	1.105 (0.774–1.641)	0.620	1.310 (0.872–1.967)	0.193	1.367 (0.910–2.054)	0.132

Model 1: univariate analysis. Model 2: Model 1 adjusted on age, gender, BMI, mean blood pressure, current smoking, diabetes mellitus, eGFR, and log-transferred albuminuria. Model 3: Model 2 with additional adjustment for total cholesterol and HDL-C (for analyses with SAA, antioxidative activity, paraoxonase activity and Lp-PLA_2_ activity as exposition variable), respective logSAA (for analyses with HDL-C and effective HDL-C as exposition variable). Model 4: Model 3 with additional adjustment for CRP. Boldface type indicates significance (*P* < 0.05).

Similarly, when considered as categorized variables, patients in the highest tertile of SAA depicted a nearly twofold higher risk for the primary endpoint {hazard ratio (HR) 1.633; 95% CI [1.099–2.428]} in univariate analysis when compared with patients in lowest tertile. This association remained significant after adjustment for traditional cardiovascular and renal risk factors (HR 1.512; 95% CI [1.007–2.269], model 2), as well as after further adjustment for total cholesterol and HDL-C (HR 1.587; 95% CI [1.053–2.392], model 3). However, after further adjustment for CRP, higher tertiles of SAA did no longer predict adverse outcome (HR 1.123; 95% CI [0.673–1.873]).

Lower tertiles of HDL-C and paraoxonase activity were predictors of cardiovascular endpoints in univariate analysis, but lost significance after adjustment for traditional cardiovascular and renal risk factors (model 2). Antioxidative property and Lp-PLA_2_ activity were not associated with cardiovascular events in any model of the Cox analyses.

## DISCUSSION

While CKD patients have a high CVD burden (29), their relative distribution of cardiac and vascular disease differs markedly from the general population (29). Many cardiovascular events in CKD patients are “nonatherosclerotic,” including sudden cardiac death and cardiac decompensation. In accordance, unlike in the general population, treatment strategies that target traditional atherosclerotic risk factors do not consistently lower cardiovascular event rates (30, 31). Thus, the establishment of novel, “nontraditional” targets for therapeutic interventions is an unmet medical need in cardiorenal medicine.

HDL-C is a robust biochemical predictor of CVD risk in the general population (32). In contrast, plasma levels of HDL-C are not associated with cardiovascular events in CKD patients (33, 34). Interestingly, increasing serum HDL-C over time is paradoxically associated with worse outcomes in incident hemodialysis patients, underlining the shortcomings of serum HDL concentrations in predicting outcomes (35). More recent data reinforced the principle of HDL “quality” in atherosclerosis that refers to the functionality of the HDL particle, as defined by its protein and lipid content, rather than HDL-C levels in plasma (9, 14, 15). Importantly, HDL composition and function are markedly affected and modified by uremia and uremia-associated inflammation (12, 13, 36) and might therefore be superior predictors of cardiovascular risk.

Recent studies by us (including predialysis patients) (17) and others (including end-stage renal disease patients on dialysis) (18) have provided clear evidence that a key function of HDL—namely, cholesterol efflux capacity—is not a predictor of cardiovascular outcome in CKD patients. This is in sharp contrast to the general population and to individuals at high risk for cardiovascular events, where cholesterol efflux capacity strongly predicts cardiovascular events (14–16).

In the present study, we tested whether other compositional and functional properties of HDL predict cardiovascular outcome among 526 nondialysis CKD patients of the CARE FOR HOMe study. We measured HDL-associated proinflammatory SAA and assessed metrics of HDL function, including activities of Lp-PLA_2_ and paraoxonase activity. CKD patients with diabetes mellitus showed lower HDL-C levels, increased SAA, and lower activities of paraoxonase and HDL-associated Lp-PLA_2_, whereas antioxidative activity was not affected. These data suggest that diabetes mellitus further affects HDL properties in CKD patients.

In univariate Cox regression analyses, lower HDL-C and lower paraoxonase activity, but also higher SAA, were predictors of adverse outcome. After adjustment for traditional cardiovascular and renal risk factors, only SAA remained a significant predictor of the primary endpoint, but did not persist after further adjustment for the systemic inflammation marker CRP.

Our results appear to be contrary to a previous study reporting that SAA predicts all-cause mortality in end-stage renal disease patients (37). However, in the respective study (37), SAA levels were not adjusted for CRP; therefore, this association may not be independent either.

The robust correlation of SAA with CRP levels in predialysis patients underlines the strong link between inflammation and the formation of SAA-enriched HDL. During the inflammatory response, SAA can displace HDL-associated apoA-I, and in extreme circumstances, SAA can account for up to 80% of the HDL protein moiety (38, 39). The contribution of HDL-bound SAA to HDL dysfunctionality is still a matter of debate. Several previous studies suggested that the presence of SAA in isolated HDL affects cholesterol efflux capacity and antiinflammatory activity of HDL (12, 13, 40–43). However, only when SAA constitutes at least 50% of total protein in HDL, the ability of HDL to remove cholesterol from human macrophages is lowered (44). Such high SAA levels were not observed in our study subjects. In line, SAA levels did not associate with metrics of HDL function. Interestingly, a recent study pointed to the hypothesis that SAA might even be protective. In American Indians with T2D and early CKD, higher levels of SAA were associated with a lower risk of end-stage renal disease (45). Moreover, a recent study provided evidence that free SAA sequesters lipid hydroperoxides and delays lipoprotein oxidation (46). Therefore, one may assume that moderate increased SAA levels observed in our study are markers rather than mediators of inflammation.

Several CKD-specific alterations in HDL metabolism may affect or even mask the association of HDL functionality with kidney function and cardiovascular events. There is evidence that *i*) conversion of pre-β-HDL to α-migrating HDL is severely impaired in CKD patients (47) associated *ii*) with an increased relative proportion of lipid poor HDL to total HDL (48). Most importantly, lipid-poor HDL and creatinine levels strongly correlate across the spectrum of CKD (48). Notably, lipid-poor HDL and small HDL particles are the most efficient acceptors of cholesterol (49) and show the highest antioxidative capacity (4). The strong correlation of lipid-poor HDL/small HDL with creatinine levels (48) may explain the unexpected inverse associations of kidney function with HDL-C efflux capacity. It also might contribute to the surprising inverse association with antioxidative activity of apoB-depleted serum. Given that the antioxidative capacity of apoB-depleted serum is predominantly determined by albumin together with low-molecular-weight hydrophilic antioxidants in addition to HDL, a reduced clearance of hydrophilic antioxidants in addition to the increase in lipid-poor HDL/small HDL might explain the inverse association of antioxidative capacity of apoB-depleted serum with kidney function.

There are several reasons why we decided to assess metrics of HDL function in apoB-depleted serum. A general problem of isolating of HDL by ultracentrifugation is that a fraction of small lipid-poor HDL particles are lost during the isolation procedure. Moreover, the dissociation and redistribution of Lp-PLA_2_ (and potentially other HDL-associated enzymes/proteins) among lipoproteins during ultracentrifugation was reported (50). We assessed cholesterol efflux capacity by using a previously validated assay that quantifies the capacity of apoB-depleted serum to accept radiolabeled cholesterol from J774 macrophages (26). Using this validated assay makes it easier to bring our results in context with other studies.

Some limitations have to be noted. To study the antioxidant properties, HDL has to be isolated by ultracentrifugation followed by size-exclusion chromatography to remove albumin and other proteins with antioxidative capacity that are coisolated with HDL (51). Isolated HDL then has to be stored frozen in the presence of cryoprotectants to avoid storage-induced loss of HDL structure and function (52). ApoB-depleted serum is more stable, and functional metrics of HDL can be measured after prolonged storage (14). Unfortunately, not enough serum was available from patients to isolate HDL.

A further limitation is that our participants were Caucasian. As the eGFR distribution may differ considerably between different ethnic groups, our results cannot be generalized to populations of other ethnic origins. Moreover, we predefined the primary endpoint, which is a composite of atherosclerotic cardiovascular events, including death of any cause. However, non-cardiovascular-related causes of death were less than 20% in our CARE FOR HOMe study (17); therefore, we can exclude a major impact on study results.

Strengths of our study include detailed assessment of multiple HDL-related activities in a nondialysis CKD clinical trial population, the prospective adjudication of all trial endpoints, and the comprehensive clinical characterization of the participants at study initiation that made it possible to adjust for potential confounding variables. As a further strength, CARE FOR HOMe is a contemporary cohort of patients across the CKD spectrum under nephrological care following evidenced-based treatment recommendations.

In conclusion, our results suggest that neither HDL quantity nor HDL quality predict future cardiovascular events in nondialysis CKD patients.

## Supplementary Material

Supplemental Data
