# Supplementary material for: HDL functionality and cardiovascular outcome among nondialysis chronic kidney disease patients
Source: J Lipid Res. 2018 May 22;59(7):1256–65. doi: 10.1194/jlr.P085076 (PMC6027904; doi:10.1194/jlr.P085076)
Supplement: Supplemental Data [file supp_59_7_1256__index.html]

HDL functionality and cardiovascular outcome among non-dialysis chronic kidney disease patients — HDL functionality and cardiovascular outcome among nondialysis chronic kidney disease patients — Supplemental Data 

# HDL functionality and cardiovascular outcome among nondialysis chronic kidney disease patients

## Supplemental Data

- Supplemental Figure S1 (.pdf, 257 KB) - Metrics of apoB-depleted serum and HDL composition and function among non-diabetic and diabetic study participants
- Supplemental Figure S2 (.pdf, 306 KB) - Kaplan Meier analyses with subsequent log-rank test
- Supplemental Figure S3 (.pdf, 312 KB) - Kaplan Meier analyses with subsequent log-rank test
