# Supplementary material for: HDL functionality and cardiovascular outcome among nondialysis chronic kidney disease patients
Source: J Lipid Res. 2018 May 22;59(7):1256–65. doi: 10.1194/jlr.P085076 (PMC6027904; doi:10.1194/jlr.P085076)
Supplement: Supplemental Data [file 10.1194_P085076_jlr.P085076-1.pdf]

Supplemental Figure S1.

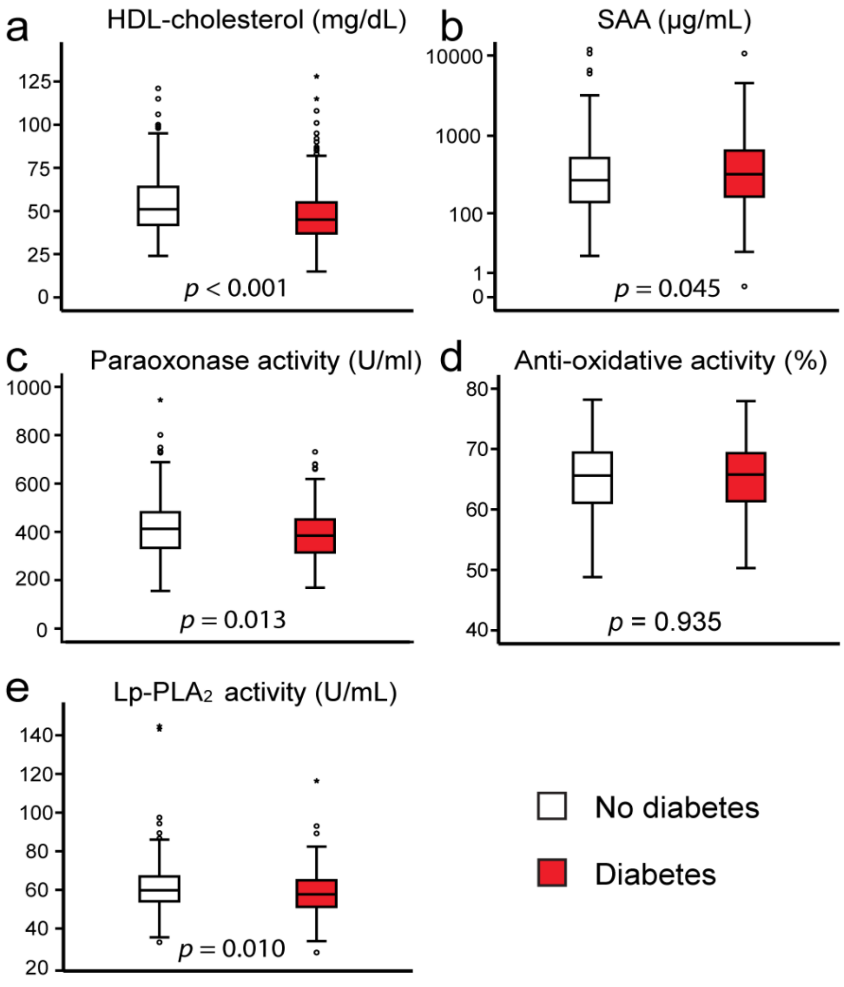

Supplemental Figure S1. Metrics of apoB-depleted serum and HDL composition and function among non-diabetic and diabetic study participants. Levels of (**a**) high-density lipoprotein-cholesterol (HDL-C), (**b**) serum amyloid A (SAA), (**c**) paraoxonase activity, (**d**) anti-oxidative activity and (**e**) lipoprotein-associated phospholipase A_2 activity (Lp-PLA_2) stratified by the presence of diabetes mellitus. Depicted are medians, interquartile ranges, and outliers.
