# Supplementary material for: HDL functionality and cardiovascular outcome among nondialysis chronic kidney disease patients
Source: J Lipid Res. 2018 May 22;59(7):1256–65. doi: 10.1194/jlr.P085076 (PMC6027904; doi:10.1194/jlr.P085076)
Supplement: Supplemental Data [file 10.1194_P085076_jlr.P085076-3.pdf]

Supplemental Figure S3.

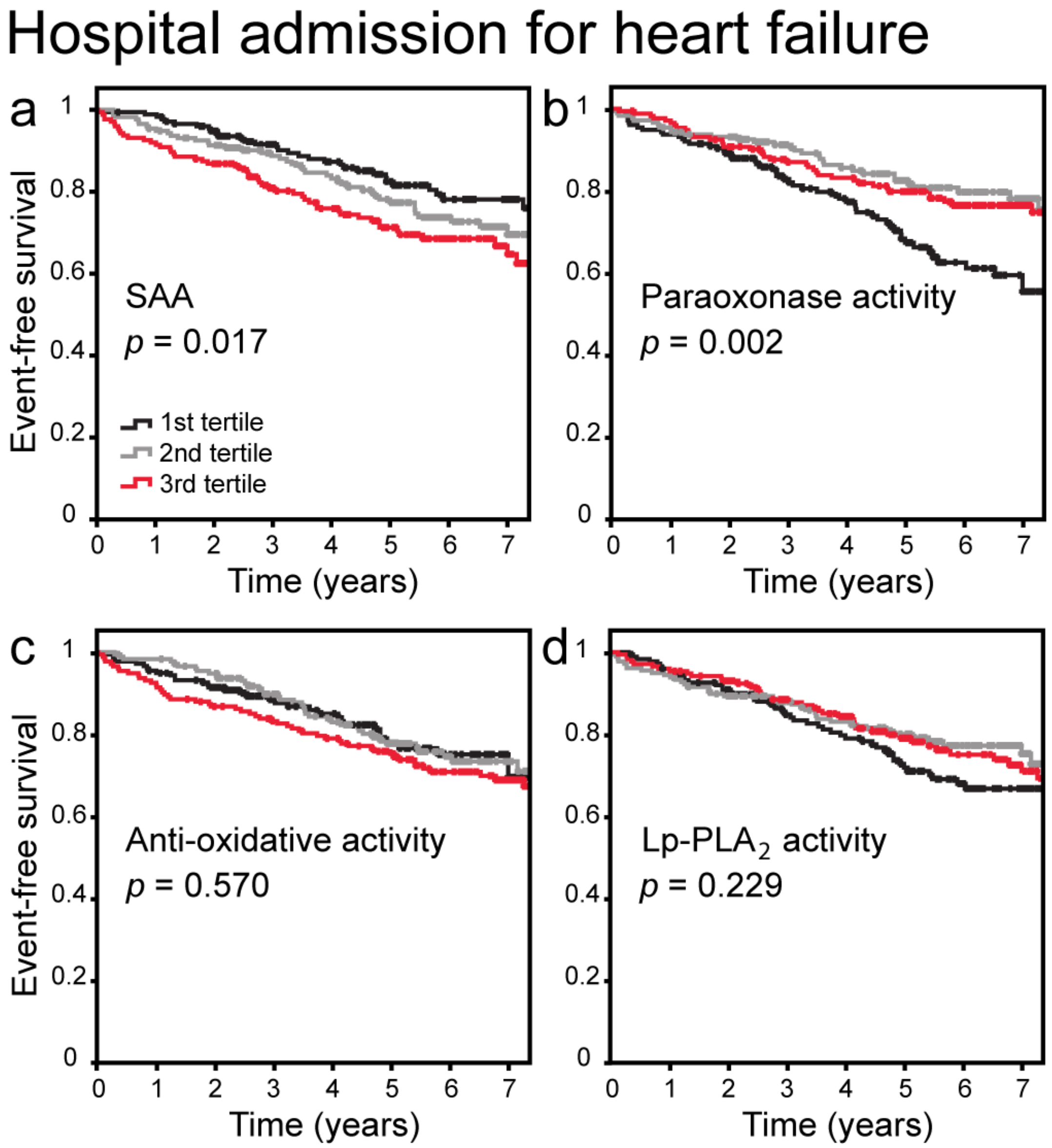

Supplemental Figure S3. Kaplan Meier analyses with subsequent log-rank test (end-point hospital admission for heart failure – event-free survival in chronic kidney disease patients) stratified by: (**a**) serum amyloid A (SAA), (**b**) paraoxonase activity, (**c**) anti-oxidative activity and (**d**) lipoprotein-associated phospholipase A_2-activity (Lp-PLA_2).
